# Microbial respiration, but not biomass, responded linearly to increasing light fraction organic matter input: Consequences for carbon sequestration

**DOI:** 10.1038/srep35496

**Published:** 2016-10-18

**Authors:** Yichao Rui, Daniel V. Murphy, Xiaoli Wang, Frances C. Hoyle

**Affiliations:** 1 Soil Biology and Molecular Ecology Group, School of Earth and Environment and Institute of Agriculture, The University of Western Australia, Crawley, WA 6009, Australia; 2 College of Agriculture, Guizhou University, Guizhou 550025, China; 3 Department of Agriculture and Food Western Australia, South Perth, WA 6151, Australia

## Abstract

Rebuilding ‘lost’ soil carbon (C) is a priority in mitigating climate change and underpinning key soil functions that support ecosystem services. Microorganisms determine if fresh C input is converted into stable soil organic matter (SOM) or lost as CO_2_. Here we quantified if microbial biomass and respiration responded positively to addition of light fraction organic matter (LFOM, representing recent inputs of plant residue) in an infertile semi-arid agricultural soil. Field trial soil with different historical plant residue inputs [soil C content: control (tilled) = 9.6 t C ha^−1^ versus tilled + plant residue treatment (tilled + OM) = 18.0 t C ha^−1^] were incubated in the laboratory with a gradient of LFOM equivalent to 0 to 3.8 t C ha^−1^ (0 to 500% LFOM). Microbial biomass C significantly declined under increased rates of LFOM addition while microbial respiration increased linearly, leading to a decrease in the microbial C use efficiency. We hypothesise this was due to insufficient nutrients to form new microbial biomass as LFOM input increased the ratio of C to nitrogen, phosphorus and sulphur of soil. Increased CO_2_ efflux but constrained microbial growth in response to LFOM input demonstrated the difficulty for C storage in this environment.

Microbial degradation of soil organic matter (SOM) is central to the carbon (C) balance in soil as SOM is biochemically processed resulting in both C stabilisation in organo-mineral complexes and C loss as atmospheric CO_2_ via microbial respiration^1^. Rather than by direct deposition of recalcitrant plant materials, stable SOM is formed predominantly as microbial detritus becomes stabilised on mineral surfaces^2^,^3^,^4^. Improved analytical techniques have confirmed that the majority of mineral-bound SOM is derived from microbial products^3^,^5^,^6^, with a layering of SOM on clay particles where proteins and polysaccharides from microbial residues aid in additional mineral-SOM stabilisation^7^. Therefore, the microbial pathway of organo-mineral formation has been increasingly recognised as the principal mechanism for long-term C accumulation^3^,^4^. However, apart from contributing to C stabilisation through microbial detritus, microbial processing of SOM will release CO_2_ through respiration which contributes to atmospheric CO_2_ and thus global climate change.

Microbial C use efficiency (CUE), which reflects the capacity of soil microorganisms to form new biomass (cell growth) rather than respiration, is a function of numerous factors including temperature and moisture, substrate availability, nutrient stoichiometry, and the physiological state of the microorganisms^8^,^9^. As a representation of decomposer C metabolism, microbial CUE defines how decomposers balance anabolic and catabolic reactions^8^. Environmental variables such as temperature can directly alter microbial metabolism and change the relationship between microbial growth and respiration - as respiration increases more than growth as a function of temperature^10^, while SOM substrate availability and quality control microbial growth rate. In soils with low labile C availability, the CUE of the microbial community is often assumed to be low; as limited C substrate is used to satisfy energy demands for cell maintenance with little left for growth and division^9^. Recalcitrant SOM substrates may decrease CUE by increasing the cost of extracellular and intracellular catabolism^11^. Stoichiometric requirement of microbial biomass for nutrients [in particular nitrogen (N), phosphorus (P) and sulphur (S)] is another controlling factor. The stabilised ‘fine fraction’ of SOM contains ~90, 19 and 14 parts of N, P and S per 1000 units of C respectively, which was more similar to the nutrient ratios found in soil microorganisms (~250, 49 and 26, and ~103, 11 and 9 parts of N, P and S biomass per 1000 units of C in bacterial and fungal, respectively) than the plant residues (~17, 2 and 3 parts of N, P and S per 1000 units of C)^12^. The ratios of these elements appear to be tightly controlled and relatively consistent across different soils and ecosystems^10^. Understanding how soil microbial partitioning of SOM substrates between respiration and growth respond to management practice associated with variable SOM substrate level is necessary to design sustainable strategies for ecosystem management to promote soil C sequestration.

Crop residues such as wheat stubbles are a global resource with potential for contributing to soil C stocks. However, many studies have indicated that soil C content does not necessarily increase as expected in response to repeated addition of large quantities of crop residues^13^,^14^. Although crop residue input into soil has often been reported to promote both microbial biomass and respiration, the magnitudes of these responses are often not proportional^15^,^16^,^17^. Therefore, the difficulty in increasing soil C by crop residue input might stem from decreased microbial CUE. Insufficient inorganic nutrients to form new biomass is another reported issue that may cause microbial CUE to decline in soils with C-rich residue input^18^. A short-term laboratory study of four diverse agricultural soils (clay range 8–60%) indicated that the capacity to generate new soil C from crop residues was mediated to a large extent by inorganic nutrient availability^19^. However, most studies report changes in microbial biomass and respiration to crop residue inputs in the field over a comparatively longer period (>one year), while short-term response to variable amount of residue input under controlled environment were rarely reported. In coarse-textured soils where physical and chemical protection of SOM is low (and respiratory activity of microbial population is maximised under optimal temperature and moisture conditions^20^) microbial respiration should increase linearly with the addition of increased amounts of fresh organic matter; while the response of the microbial biomass may not be linear due to the dependence on nutrient availability for growth. To date, studies of the short-term response in microbial respiration and biomass to the application of fresh organic matter level is lacking in soils with comparatively different organic matter backgrounds.

Agricultural systems of semi-arid Western Australia are typified by low rainfall and high temperature, with predominately coarse-textured infertile soils that support low crop yields (<2 t ha^−1^ wheat grain yield)^21^, limiting the amount of plant residue input to the soil^22^. Light fraction organic matter (LFOM), which represents plant-derived organic matter, is sensitive to environmental variables and management practices^23^. Obtained through density fractionation, the LFOM is partly decomposed and is relatively more homogeneous than raw plant residues^23^. Within this semi-arid environment management practices such as stubble retention, minimum tillage and crop-pasture rotation have little impact on total SOM but can increase LFOM concentration^24^,^25^,^26^.

Here we investigated the soil C sequestration potential of agricultural systems of semi-arid Western Australia through Roth-C modelling and a laboratory incubation experiment. Soils from a trial site which had received long-term (10 years) external plant residues input were used. Modelling was conducted to determine if soils at this site were C-saturated or still had the potential to store more C. Then in a laboratory incubation we investigated how microbial properties (respiration, biomass and C use efficiency) and soil physico-chemical properties responded to a gradient of LFOM addition and field SOM level. In particular, we monitored the metabolic quotient *q*CO_2_ [CO_2_-C/Microbial Biomass C (MBC)] and the microbial quotient (MBC/soil C) to explore microbial CUE. We also measured extracellular enzyme activity for β-glucosidase and cellulase as they catalyse different steps of SOM decomposition and reflect the degradation of labile or recalcitrant compounds. We hypothesised that: (1) both microbial respiration and biomass would respond positively to increasing rates of LFOM input in coarse-textured soil; and (2) adding LFOM would benefit N cycling and enzyme activities in soils both with or without long-term external plant residue inputs.

## Results

Roth-C modelling (see Supplementary Fig. S1a) suggests that the field treatment without additional plant residues (tilled) was not yet C saturated (100 year attainable soil total C ~30 t C ha^−1^; 0–30 cm) with additional capacity to store more C. Under current cropping systems where 80% of stubble is retained in a low yielding environment (wheat grain yield 1.5 t ha^−1^ based on achieving 46% of water limited potential yield) and where 50% of C from 0 to 30 cm is located in the top 10 cm layer of soil; C stored in soil from 0 to 30 cm has reached approximately 60% of the attainable level based on climate and soil type^24^. In comparison, the treatment receiving additional inputs of plant residues (tilled + OM) suggest that soil total C is nearing its attainable storage for this specific farming system – but the long-term outlook suggests the majority of this is relatively short lived (see Supplementary Fig. S1b) with no additional C storage evident were inputs to cease (see Supplementary Fig. S1b).

Field trial soil with different historical plant residue inputs [total C content: control (tilled) = 9.6 t C ha^−1^ versus tilled + plant residue treatment (tilled + OM) = 18.0 t C ha^−1^] were incubated in the laboratory with a gradient of LFOM (0.068, 0.169, 0.338, 0.676 and 1.690 g per 200 g^−1^ dry weight soil) added into separate soil samples to modify soil C content. This was equivalent to 20%, 50%, 100%, 200% and 500% of the original LFOM content of the tilled soil. Results showed that increased total soil C derived from either historic field amendments of plant residues (80 t additional dry matter ha^−1^ over 10 years), or from the recent addition of LFOM in the laboratory study had a significant influence on soil physico-chemical properties (Table 1). Plant residue amendment significantly increased (*p* < 0.001) total C in field soil (tilled + OM), which was nearly twice that of the tilled (control) soil (Table 1 and S1). After 12 weeks’ incubation, in soils that had different historical plant residue input and recent addition of LFOM, a positive correlation with total C was observed for both WHC (y = 1.37x + 28.0, R^2^ = 0.96, *p* < 0.001, n = 48) and CEC (y = 0.41x + 0.45, R^2^ = 0.97, *p* < 0.001, n = 48).

In field soils WHC increased by 25% (*p* < 0.001) with historic amendments of plant residues (tilled + OM) compared to control soils receiving no additional plant residues. Associated with the change in total C, a larger CEC (approximately 70% relative increase; *p* < 0.001) was also observed in field soils with added plant residues than in control soils. Increasing rates of LFOM amendment in short-term incubations also resulted in an increase in CEC (*p* < 0.001). Results for background soils with no added LFOM indicate that for this site the relationship between total C and measured variables was stable. A 1% change in actual total C would increase WHC by the equivalent of 13.8 mm water 100 mm^−1^ depth and CEC by 3.1 meq 100 g^−1^ (Table 1). This is similar to the values calculated for soils with more recently added LFOM (WHC 13.5 mm 100 mm^−1^ depth; CEC 4.1 meq 100 g^−1^ dry weight soil). In contrast to WHC and CEC, soil pH declined in both field soils receiving additional plant residues and with the addition of LFOM to short-term incubated soils (y = −0.03x + 6.98; R^2^ = 0.96; *p* < 0.001).

**Figure 1:**
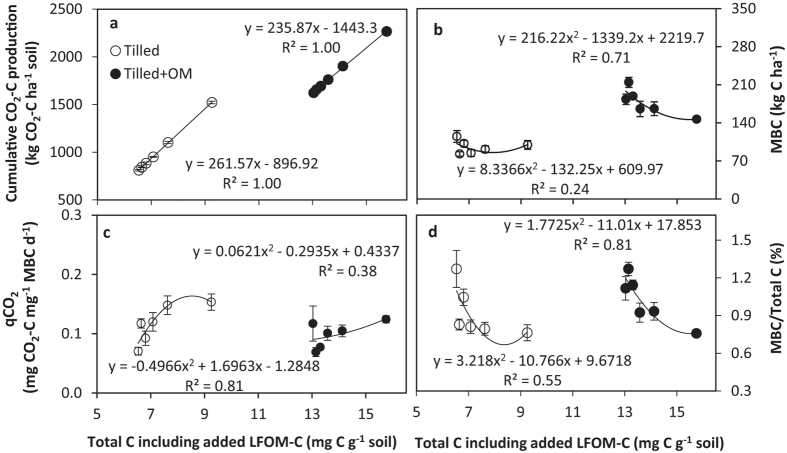
(**a**) Cumulative CO_2_-C production from the 12-week laboratory incubation, (**b**) microbial biomass C (MBC), (**c**) metabolic quotient *q*CO_2_, and (**d**) microbial quotient MBC/total C in soils with different field soil organic matter (SOM) level and variable rates of light fraction organic matter (LFOM) amended soil that was incubated at 45% water holding capacity at 25 °C measured over 12 weeks. Data (except 12 week cumulative CO_2_-C production) measured after 6 weeks and 12 weeks were averaged. Bars represent the standard error of the mean (n = 4 reps).

Microbial respiration as determined by the average total CO_2_-C loss during a 12 week incubation was the equivalent of 0.97 t C ha^−1^ for tilled soils (0.77 to 1.45 t C ha^−1^) and 1.84 t C ha^−1^ for the tilled + OM soils (1.65 to 2.30 t ha^−1^; Fig. 1a). Both higher field soil total C content (*p* < 0.001) and LFOM addition (*p* < 0.001) significantly increased CO_2_-C efflux, but no interaction between the two factors was found (*p* = 0.84). Microbial respiration (CO_2_-C) showed a linear response to increased LFOM inputs (R^2^ > 0.99, Fig. 1a). The field SOM status did not influence the LFOM induced CO_2_-C loss (LFOM treatment minus control), with the equivalent of approximately 20% of the added LFOM-C across all LFOM additions being respired from both tilled and tilled + OM soils.

Microbial biomass C was higher in tilled + OM soils (*p* < 0.001; 356 kg C ha^−1^) than in the tilled soils (172 kg C ha^−1^) for pre-incubated soils (Day 0, Table 1). Therefore of the additional 36 t C ha^−1^ (80 t ha^−1^ chaff) added to tilled + OM plots over 10 years, 23% was retained as soil C and 5% was converted to MBC. Microbial biomass C decreased during the incubation and after 12 weeks measured 183 kg C ha^−1^ in tilled + OM soils and 115 kg C ha^−1^ in tilled soils (Fig. 1b). The addition of LFOM caused a significant decline (*p* < 0.004) in MBC (see Supplementary Table S1) which was more evident in the tilled + OM soil, which decreased from 183 kg C ha^−1^ in the control (no added LFOM) to 147 kg C ha^−1^ in the soils with five times the background level of LFOM addition (Fig. 1b). There was a significant interaction (*p* = 0.001) between background soil C level and recent addition of LFOM on MBC (see Supplementary Table S1) which suggested the response to LFOM was dependent on the background C level of the soils (Fig. 1b). The microbial quotient (MBC/soil C) decreased with LFOM addition (*p* < 0.001), while the metabolic quotient *q*CO_2_ (CO_2_-C/MBC) increased with LFOM addition (*p* < 0.001). This contrasted with the observations for field soils with no added LFOM - the tilled + OM soils that had inherently higher background C showed both a lower metabolic and higher microbial quotient (*p* < 0.05) than tilled control soil where there had been no additional plant residue inputs (Fig. 1c,d). A significant interaction was also evident between soil C background and LFOM (*p* = 0.019) for the metabolic quotient *q*CO_2_ (CO_2_-C/MBC), with lower efficiency evident in soils with high background C and no added LFOM and a general increase in efficiency which was only significant at 20% and 200% addition for these soils relative to tilled soils with added LFOM (Fig. 1c). These results suggested that historical input of plant residues resulted in higher microbial CUE, whereas increasing amounts of new LFOM input decreased CUE.

**Figure 2:**
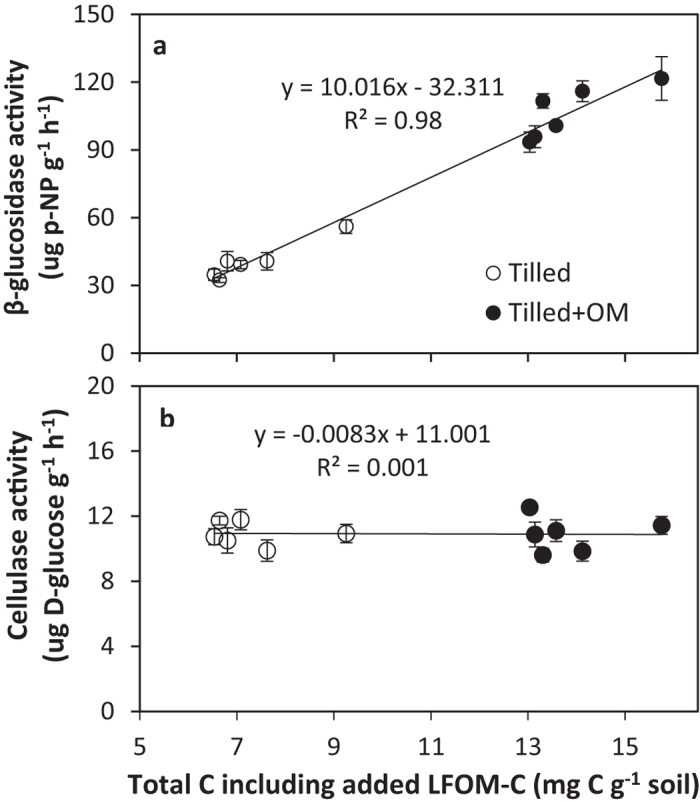
(**a**) β-glucosidase (μg p-NP g^−1^ h^−1^) and (**b**) cellulase activity (μg D-glucose g^−1^ h^−1^) measured in soils following incubation of soils with different field soil organic matter (SOM) level and variable rates of light fraction organic matter (LFOM) at 45% water holding capacity and 25 °C. Data is the average of two sampling times at 6 and 12 weeks. Bars represent the standard error of the mean (n = 4).

Over the incubation period β-glucosidase activity decreased (*p* < 0.001), while cellulase activity increased (*p* < 0.001). Increased β-glucosidase activity was associated with higher field soil C content (tilled + OM; 181 μg p-NP g^−1^ h^−1^) when compared to the control soil (tilled; 71 μg p-NP g^−1^ h^−1^; *p* < 0.001). Short-term incubation decreased β-glucosidase activity by approximately 50% in soils where no LFOM was added. Light fraction organic matter addition significantly increased β-glucosidase activity (*p* < 0.001). β-glucosidase (y = 10.0x − 32.3; R^2^ = 0.98, *p* < 0.001) and acid phosphatase (y = 23.5x + 9.3; R^2^ = 0.93, *p* < 0.001) activity showed a strong positive correlation to total C irrespective of the organic matter source, accounting for 69% and 61% of the variation in individual activity measures explained respectively (P < 0.001, n = 104). In contrast, cellulase activity was not significantly (*p* > 0.05) influenced by background soil C level and showed a small but variable response to LFOM addition on (Fig. 2b).

**Figure 3:**
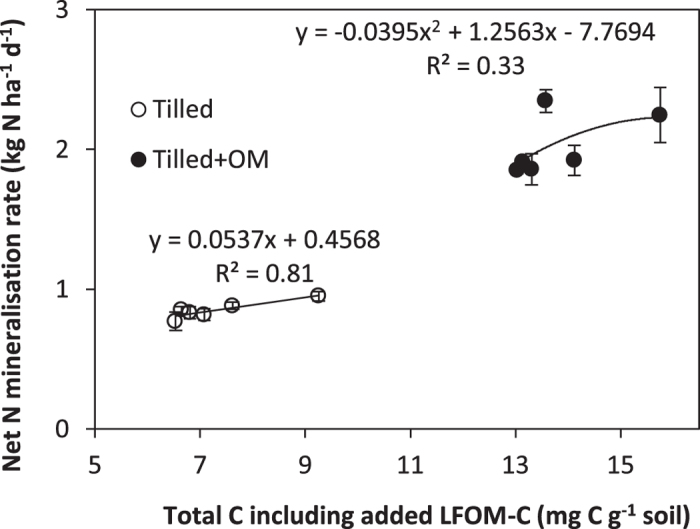
Net nitrogen (N) mineralisation rate (kg N ha^−1^ d^−1^) of soils with different field soil organic matter (SOM) level and variable rates of light fraction organic matter (LFOM) amended soil incubated at 45% water holding capacity and 25 °C for 12 weeks. Bars represent the standard error of the mean (n = 4).

Inorganic N (NO_3_^−^ + NH_4_^+^) concentration increased (*p* < 0.001) in soils with higher background SOM (Table 1), the majority of which was present as nitrate (NO_3_^−^). Net N mineralisation rates were higher in soils with a greater amount of field total C content (*p* < 0.001) and increased with short-term incubation after LFOM amendment (*p* < 0.01; Fig. 3) in both control (R^2^ = 0.81, *p* < 0.001) and tilled + OM treatments (R^2^ = 0.33, *p* < 0.001).

## Discussion

Roth-C modelling suggests that the field soils had not reached their attainable level of C sequestration, indicating additional capacity to store a proportion of the newly applied LFOM-C. Although adding LFOM to soil increased short-term soil C, the additional LFOM input also resulted in C loss through microbial respiration after input ceased without promoting microbial growth (Fig. 1). This demonstrated the difficulty of increasing total soil C stock through external crop residue input in coarse-textured soils. The amount of C that is ultimately stabilised depends on both the soil’s capacity for organic matter stabilisation^27^,^28^ and how effectively the microbial community converts plant-C to biomass^29^. Soil organic matter in coarse-textured soils is more vulnerable to decomposition due to reduced potential for C stabilisation in aggregates or organo-mineral complexes^30^. Coarse-textured soils usually have more active microbial population than fine-textured soils^20^,^31^. Mtambanengwe *et al*.^32^ also reported that decomposition of added organic matter was governed by its physical accessibility by microorganisms as determined by soil texture and pore size distribution, and pores of <75 μm were responsible for protection of organic substrates against microbial decomposition in soil. As a result, large pore sizes in this coarse-textured soil would favour decomposition and CO_2_-C loss because soil microorganisms could easily access the added labile C substrate. Apart from the physico-chemical environment of soil, the SOM decomposition is also determined by the type and quality of the plant residue or organic input^33^. Unlike the physically and chemically protected SOM pools, the unprotected LFOM pool is more easily decomposed^34^. Therefore, increasing the amount of LFOM input resulted in a linear increase of CO_2_-C emission in these coarse-texture soils. The metabolic quotient increased with increasing rates of recent LFOM input. The constrained microbial biomass and microbial quotient in response to increasing LFOM input in this study might be attributed to insufficient inorganic nutrients to support stoichiometric ratio of C, N, P and S. The LFOM used in this study contained ~56, 3, and 5 parts of N, P and S per 1000 units of C, as compared to ~90, 19 and 14 parts per 1000 units in the stabilised ‘fine fraction’ of SOM. Cayuela *et al*.^35^ suggested that N was a clear limiting factor for microbial growth when crop residues were added. In a 28 day incubation they found rapid N (ammonium) immobilisation in soil with crop residue input. Kirkby *et al*.^19^ also showed that the capacity to generate ‘new’ soil C from C-rich crop residues in a range of soils is mediated to a large extent by the availability of inorganic nutrients. As such, the long-term sequestration of C from crop residues into SOM will require the simultaneous sequestration of significant amounts of nutrients in order to meet the stoichiometric requirements of the soil microbial biomass and newly generated SOM. Therefore, the management of inorganic nutrients is an important consideration for strategies to build soil C from crop residues, and how this might be achieved economically is a challenge.

Soil enzymes catalyse SOM decomposition and nutrient cycling processes and are regarded as important indicators of soil fertility^36^. Over the incubation period, MBC and β-glucosidase activity decreased, while cellulase activity increased. This might suggest that the microbial community was shifting from r-strategists towards K-strategists as easily decomposable substrates were exhausted^37^. The activity of β-glucosidase may reflect the initial rapid decomposition of the more labile SOM fraction, for instance glucose production. Cayuela *et al*.^35^ also found an immediate and remarkable increase in β-glucosidase activity within 2 days of wheat straw addition, which decreased thereafter. The fast-growing r-strategists that mainly live on labile C often die quickly after the exhaustion of easily available substrate. By contrast, the K-strategists grow much more slowly and steadily on stabilised SOM fractions and are responsible for the degradation of more complex fractions such as cellulose and lignin^35^. The released biomass of r-strategists can be used as energy source or reassimilated for microbial growth of K-strategists^38^.

Strong correlation between soil total C and activity of β-glucosidase (R^2^ = 0.95) showed that the input of crop residues favoured the cycling of simple sugars and glucose production (Fig. 2). However, no response of cellulase activity to historical input of crop residue and recent addition of LFOM suggested that the decomposition of more complex compounds (by K-strategists) was slow and unaffected by these treatments. Polymerised compounds such as lignin and cellulose persist in soils longer than simple substrates, while 40 to 60% of enzyme activity comes from stabilised enzymes^36^. Light fraction organic matter inputs increased net N mineralisation significantly in the present study, reflecting its role as a short-term nutrient reservoir (Fig. 3). Our data agree with those of Cookson and Murphy^39^ who reported that LFOM had a major influence on microbial mediated N mineralisation–immobilisation turnover in coarse-textured agricultural soils. However this increase in NO_3_^−^ concentration did not alleviate the nutrient stress on microbial biomass in the short term; possibly because this slow release of nutrients did not meet the requirement of microorganisms for nutrients to process the C-rich LFOM input. Soil heterotrophs generally prefer NH_4_^+^ rather than NO_3_^−^ for their growth^35^ but in these soils NH_4_^+^ is either efficiently taken up by microorganisms or rapidly nitrified to NO_3_^−^ which accumulates in soils with higher LFOM input and activity of enzymes (e.g. β-glucosidase). However, this slow release of N might be of long-term benefit to the microbial biomass^27^, which may explain the greater microbial biomass in soils with historical crop residue input (tilled + OM) than control (tilled) when no additional LFOM was applied.

Soil organic matter helps to create and stabilise soil pores, promotes the formation of soil aggregates and contributes to soil water storage via its capacity to absorb water. Our data are in line with Angers and Carter^40^ who also suggested that labile C is positively related to macro-aggregate stability and contributes to WHC of soil. Soil organic matter contributes significantly to the CEC of soils low in clay content and their subsequent ability to retain nutrients. In this study we show a 1% change in soil C is associated with CEC change of 3.1 meq 100 g^−1^, which is considerably more than an estimated CEC change of 0.7 meq 100 g^−1^ for a 1% increase in clay for this environment. In the absence of non-wetting constraints the broadacre application of clay as an ameliorant is likely unprofitable. Similarly the profitability and practicality of applying large inputs of organic matter from off-site is unrealistic. However long-term incremental increases in SOM inputs may prove an effective way to slowly improve nutrient retention and cycling in low clay content soils and positively influence agricultural production.

## Conclusion

Roth-C modelling showed there was further capacity for soil C storage in this semi-arid coarse-textured soil through increasing and sustaining inputs of plant residues. Long-term results between soils with differing C backgrounds confirm accumulation of SOM is achievable at this site. In practice however, increased rates of LFOM input to soil resulted in significant loss of labile C through microbial respiration and lower MBC; which we propose was due to insufficient inorganic nutrients to form microbial biomass. Our findings illustrate the difficulty of increasing the total soil C stock through extra plant residue inputs in semi-arid dryland cropping regions, while proper management of inorganic nutrients needs to be considered for further increase of soil C storage. Building soil C in this environment is challenged both in context of increasing organic inputs sustained through significant changes in net primary productivity and in retaining this C long term where little physical protection exists.

## Methods

### Field trial site and soil description

The field trial used for soil collection was located in Buntine, Western Australia (30.00° S, 116.33° E). The region has a semi-arid climate, with hot, dry summers and cool, wet winters (when cropping occurs). Based on 17 years of climate data (1997–2014) the area has a mean annual rainfall of 284.9 mm, mean monthly temperatures ranging from 5.8 to 35.3 °C and actual temperatures ranging from −1.0 to 46.9 °C (http://www.bom.gov.au/climate/data). Soil at the site was a deep sand (92% sand, 2% silt, 6% clay) and classified as a Basic Regolithic Yellow-Orthic Tenosol (Australian soil classification; Isbell, 2002), or a Haplic Arenosol (World Reference Base for Soil Resources; IUSS Working Group WRB, 2007).

### Field trial and soil collection

The field trial was established in 2003 with a three year lupin–wheat–wheat rotation and a range of field management treatments (randomised block deign) to create a range of total SOM contents. Each treatment had three field replicate plots that were 80 m long and 10 m wide. Two treatments (tilled, tilled + OM) with contrasting total C contents were used in this study. The tilled treatment (control) was cultivated to 10 cm depth annually using offset discs before seeding with knife point tines. Over a period of 9 years the tilled + OM treatment had 20 t ha^−1^ of barley (2003; C:N ratio 75:1), canola (2006; C:N ratio 60:1), oat (2010; C:N ratio 70:1) and oat chaff (2012; C:N ratio 70:1) mixed into the soil with offset discs (i.e. an additional 80 t ha^−1^ plant residues was added in addition to the annual inputs resulting from above-ground plant stubble residue returns of *ca*. 1.5–3 t ha^−1^). This represented an additional plant residue input of 36 t C ha^−1^ in the tilled + OM treatment. As a result soil total C (0–10 cm) measured 18.0 t C ha^−1^ in tilled + OM treatment and 9.6 t C ha^−1^ in tilled treatment.

Soil (0–10 cm) was collected manually using a push-in sand auger on 4^th^ June 2013. Separate composite soil samples comprising 40 cores (7 cm diameter by 10 cm depth) were collected from each of the field replicate plots using a zigzag sampling pattern. Soils collected from each of the tilled (control) and tilled + OM treatments were dried at 40 °C for 7 days prior to being sieved (4 mm sieve) and mixed thoroughly for the subsequent incubation study.

### Modelling of attainable soil C

Modelling using Roth-C model was conducted to determine if soils at this site were C saturated or still had the potential to store more C. It is possible that where soils are C saturated, resultant microbial processes and efficiency could be influenced. The Roth-C model (version 26.3) operating in Excel© was used to model attainable soil C values in non-waterlogged soils as described by Jenkinson *et al*.^41^. Roth-C has previously been validated for use in dryland agricultural systems for both crop and pasture in Australia^24^,^42^,^43^. The model was initiated by partitioning soil C into six pools - decomposable plant (DPM) and resistant plant material (RPM) pools, fast (BIOF) and slow microbial biomass pools (BIOS), humified organic matter (HUM) and inert organic matter (IOM) which included the char fraction^42^. Where possible actual pool allocations determined by mid-infrared spectroscopy^44^ from the site were used (8%, 69% and 19% for RPM, HUM and IOM respectively) - otherwise reported allocations for soil C pool values within Roth-C for Australian soils (1.7% and 0.02% for BIOF, BIOS)^45^ and remainder (2.1%) was allocated as DPM given high amounts of larger sized plant material present. Climate data (lat. –30.00, long. 116.20; 2003–2012) and soil properties used to initiate the Roth-C model for the experimental site included average monthly rainfall (mm), open-pan evaporation (mm), temperature (°C) and clay content (6.3%). Other site-dependent variables included crop rotation, plant residue inputs, percentage of stubble retained and measured actual soil C stock (t C ha^−1^) at the beginning of the experiment.

Current land-use scenarios were constructed to initialise Roth-C to determine the attainable SOC storage. A continuous cropping system (see Supplementary Table S2) was modelled for a deep sand (6% clay) as follows - assumed 7 months dry matter production from May, no grazing, and retaining 80% of plant stubble residues. A root to shoot ratio of 0.5:1 was used with production based on 20 kg grain mm^−1^ available water, 46% water use efficiency and dry matter on a harvest index of 0.37. In this example, available water was calculated as one third of growing season rainfall (equivalent to one third of rainfall from January to March inclusive, plus rainfall from April to October). Two input scenarios were modelled to reflect attainable SOC values for each representative treatment: 1. No additional plant residue inputs and 2. Four applications each of 20 t dry plant residues ha^−1^, see Supplementary Table S2).

### LFOM fractionation and incubation

Light fraction organic matter material used for the laboratory incubation study was collected from field soils (tilled, tilled + OM) using a density floatation method as described by Cookson *et al*.^25^. Briefly, soils were shaken in milli-Q water (specific density of 1.0 g cm^−3^) on a reciprocal shaker for 1 h, left to settle overnight and any LFOM floating removed by suction on to a 20 μm nylon filter and washed with milli-Q water. The recovered LFOM was then air dried (40 °C for 72 h), mixed evenly and gamma irradiated at 50 kGy before being applied to the incubated soils. The total C, N, P and S of the LFOM was 32%, 1.8%, 0.1% and 0.2%, respectively (C:N:P:S ratio of 1000:56:3:5). A previous study on Western Australian soils confirmed that LFOM (<1.0 g cm^−3^) had a similar O-alkyl:alkyl-C ratio to plant material in the early stages of decomposition^46^.

Soil samples (200 g dry weight equivalent) were weighed into air-tight, 1000 mL glass jars and sealed using lids modified with gas septum ports. Light fraction organic matter was added at the amount of 0.068, 0.169, 0.338, 0.676 and 1.690 g (representing 20%, 50%, 100%, 200% and 500% of the original LFOM content of the tilled soil) into separate soil samples, then mixed and incubated at 25 °C and 45% WHC for either 6 or 12 weeks. Control jars with 0% LFOM addition to soil were also established. Each treatment was replicated four times and duplicate sets of jars were incubated to allow for sampling and analyses at 6 and 12 weeks. After 6 weeks and 12 weeks, soils were destructively sampled from each of the two set of jars and a range of soil properties measured.

### Soil properties

CO_2_-C evolved from the soil was measured in the headspace of the jars twice a week using an infrared gas analyser (IRGA). Headspace gas samples (1 mL of air from each jar, collected after first mixing the headspace) were analysed against a CO_2_ standard [4.95% (±0.10%) CO_2_ in helium, BOC Ltd]. The CO_2_-C results presented are the average daily rate of evolution measured for each treatment, minus a control treatment (no soil) to account for the CO_2_ concentration already in the jar headspace. After each sampling, all jars were opened and the headspace gas exchanged with fresh air. Oxygen availability was determined not to be limiting for the microbial population in the sealed incubation jars. It has been reported that the soil microbial respiratory quotient [CO_2_ evolution (mL): O_2_ consumption (mL) ratio] has a range of 0.5–1.5 in basal respiration and in soils that have received organic matter substrates^47^. In the present study the CO_2_ production for all soils during all sampling periods ranged from 20–60 mL, indicating that O_2_ consumption should be in the range 13–120 mL. The O_2_ in the headspace of the 1000 mL jar was about 190 mL, greater than the O_2_ consumption for all sampling periods. Therefore, O_2_ availability was not limiting for the microbial population in the incubation jars.

Total C and N were determined on air dried, finely ground soil by total combustion using a C/N-analyser (Elementar Vario Macro CNS, Hanau, Germany). Soil ammonium (NH_4_^+^) and NO_3_^−^concentrations were determined using 20 g of fresh soil shaken in 80 mL of 0.5 M K_2_SO_4_ for 1 h and filtered (Whatman #42). Ammonium and NO_3_^−^ concentrations in the K_2_SO_4_ extracts were analysed colorimetrically by automated segmented flow auto-analysis (OI Analytical, College Station, Texas, USA). Microbial biomass C was determined by fumigation extraction using 20 g (oven-dried equivalent) of soil shaken in 80 mL of 0.5 M K_2_SO_4_ for 1 h. Non-fumigated soils were also extracted. Oxidisable C in the K_2_SO_4_ extracts was determined using an Aurora 1030 Total Oxidisable C analyser (OI Analytical, College Station, Texas, USA). A k_EC_ factor of 0.45 was used to calculate the MBC. Soil pH was determined on air dried soil (10 g) in 50 mL of CaCl_2_ (0.01 M), shaken for 1 h and left to stand overnight. Cation exchange capacity was determined by Inductively Coupled Plasma (ICP) Spectroscopy^48^. Water holding capacity of soil was determined by using a kiln slab and drying at 105 °C for 24 h^48^.

β-glucosidase was determined following a 1 h incubation of soil at 37 °C with 0.25 mL of toluene, 4 mL of universal buffer, and 1 mL of 25 mM p-nitrophenyl-β-D-glucosidase (PNG)^49^. The p-nitrophenyl concentration was determined colorimetrically using a UV/VIS spectrophotometer at 400 nm, after extraction of supernatant with 1 mL of CaCl_2_ and 4 mL of Tris (hydroxymethyl) aminomethan (THAM, pH 12). Cellulase activity was determined after incubation of 1.5 g dry weight soil with 0.5 g Avicel at 40 °C for 16 h^50^. Reducing sugars were determined colourimetrically on a UV/VIS spectrophotometer at 520 nm and adjusted using a correction factor for soil water content. The initial values of soil basic properties before the incubation were listed in Table 1.

### Statistical analyses

Repeated measures analysis of variance (ANOVA) was used to examine the effects of sampling time on these parameters (Supplementary Table 1). While small changes were noted in some measures between the 6 and 12 week sampling as a result of extended incubation, the resultant response of variables measured to either field plant residue or LFOM treatments did not change. Thus data for the 6 and 12 week sample times have been averaged. Analysis of variance was used to examine the effects of field soil C level (i.e. tilled and tilled + OM field trial soil values), recent LFOM addition (i.e. laboratory incubation treatments; 0 to 500%), and their interactions on CO_2_-C evolution, total C, total N, C:N ratio, WHC, CEC, soil pH (CaCl_2_), net N mineralisation rate, MBC, and β-glucosidase and cellulase activity. Linear regression was used to examine the relationships between the properties. All statistical analyses were performed in GenStat V16.0 (VSN International, UK).

## Additional Information

**How to cite this article**: Rui, Y. *et al*. Microbial respiration, but not biomass, responded linearly to increasing light fraction organic matter input: Consequences for carbon sequestration. *Sci. Rep.* **6**, 35496; doi: 10.1038/srep35496 (2016).

## Supplementary Material

Supplementary Information

## Figures and Tables

**Table 1:** Soil (0–10 cm) properties measured for different field treatment soils prior to laboratory incubation: total carbon (C), total nitrogen (N), soil C:N:P:S ratio, microbial biomass carbon (MBC), pH(CaCl_2_), inorganic nitrogen (NH_4_^+^, NO_3_^−^), water holding capacity (WHC), cation exchange capacity (CEC), and enzyme activity of β-glucosidase and cellulose.

Field Treatment	Total C (%)	Total C (t C ha^−1^)	Total N (t N ha^−1^)	C:N:P:S ratio	MBC (kg C ha^−1^)	pH (CaCl_2_)	Inorganic N (kg N ha^−1^)	WHC (mm 100 mm^−1^ depth)	CEC (meq 100 g^−1^)	β-glucosidase (μg p-NP g^−1^ h^−1^)	Cellulase (μg D-glucose g^−1^ h^−1^)
Tilled	0.65 (0.02)	9.6 (0.2)	0.82 (0.07)	1000: 98:17:10	172 (4)	6.8 (0.01)	24 (0.4)	36 (0.2)	3 (0.1)	71 (2)	1.4 (0.6)
Tilled + OM	1.30 (0.02)*	18.0 (0.3)*	1.44 (0.02)*	1000: 81:14:11	356 (5)*	6.7 (0.00)*	60 (1.6)*	45 (0.7)*	5 (0.4)*	181 (5)*	3.5 (0.5)*

Standard error of the mean (n = 4) is shown in parentheses and *indicates a significant difference at *p *< 0.05.
