# Supplementary material for: Microbial respiration, but not biomass, responded linearly to increasing light fraction organic matter input: Consequences for carbon sequestration
Source: Sci Rep. 2016 Oct 18;6:35496. doi: 10.1038/srep35496 (PMC5067501; doi:10.1038/srep35496)
Supplement: Supplementary Information [file srep35496-s1.pdf]

Supplementary Information

Title: Microbial respiration, but not biomass, responded linearly to increasing light fraction organic matter input: Consequences for carbon sequestration

Running head: Organic inputs influence microbial C dependent processes

Authors: Yichao Rui[1], Daniel V. Murphy[1], Xiaoli Wang[1, 2], Frances C. Hoyle[1, 3, *]

Figures

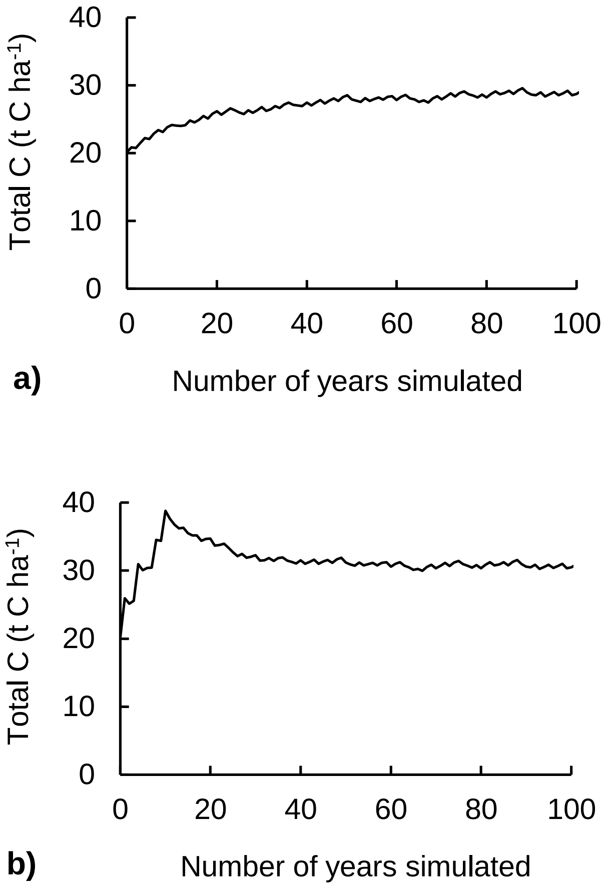

Supplementary Figure S1 Attainable soil organic carbon (t C ha^{-1}, 0–30cm) modelled for 100 years under a continuously cropped system on a deep sand (6.3% clay) at Buntine in south-western Western Australia with a) no additional external organic matter inputs (tilled control) and b) with four additional inputs each of 20 t dry plant residues ha^{-1} (tilled + OM). Modelled with Roth-C using site climate (30 00'S 116 12'E) and input data for the initial 11 years (2003

to 2013), and subsequent to this using consecutive rolling rainfall, temperature and evaporation data (from 1980 to 2013; data provided by the State of Queensland). The modelled data assumed attainable soil organic carbon values were not constrained by practical limitations to plant growth and used actual carbon values at time 0 to initialise values the model.

Tables

Supplementary Table S1 Significance levels for a repeated measures (6 and 12 week) ANOVA conducted to assess the effect of background field soil organic carbon (SOC) treatments (tilled, tilled + organic matter) and light fraction organic matter (LFOM) addition, and their interaction on total carbon (C), total nitrogen (N), water holding capacity (WHC), cation exchange capacity (CEC), soil pH ($CaCl_2$), ammonium (NH_4^+) and nitrate (NO_3^-) concentration, microbial biomass C (MBC), metabolic quotient (qCO_2), and activities of β-glucosidase and cellulase.

Factor	Total C (t C ha^{-1})	Total N (t N ha^{-1})	WHC (mm $100mm^{-1}$ soil depth)	CEC (meq $100g^{-1}$)	pH ($CaCl_2$)	NH_4^+ (kg N ha^{-1})	NO_3^- (kg N ha^{-1})	MBC (kg C ha^{-1})	qCO_2	β-Glucosidase activity (µg p-NP g^{-1} h^{-1})	Cellulase activity (µg D-Glucose g^{-1} h^{-1})
SOC background	<0.001	<0.001	<0.001	<0.001	<0.001	<0.001	<0.001	<0.001	0.169	<0.001	0.938
LFOM	<0.001	<0.001	<0.001	0.001	<0.001	0.009	0.001	0.004	0.002	<0.001	0.013
SOC×LFOM	0.341	0.903	0.151	0.100	0.328	0.030	0.033	0.001	0.019	0.424	0.177

Supplementary Table S2 Rotation management at Buntine from 2003 to 2013 on imposed treatments for the 'tilled' and 'tilled + organic matter (OM)' treatment.

Year	Crop type	Treatment notes
2003	Lupin	Brown manure lupin crop. Organic matter treatments received barley chaff spread at 20 t ha^{-1}. Both treatment tilled with offset discs.
2004	Wheat (cv. Wyalkatchem)	Both treatment tilled with offset discs.
2005	Wheat (cv. Wyalkatchem)	Both treatment tilled with offset discs.
2006	Lupins	Brown manure lupin crop. Organic matter treatments received canola chaff spread at 20 t ha^{-1}. Both treatment tilled with offset discs.
2007	Wheat (cv. Wyalkatchem)	Both treatment tilled with offset discs.
2008	Wheat (cv. Wyalkatchem)	Both treatment tilled with offset discs.
2009	Lupin	Brown manure lupin crop. Both treatment tilled with offset discs.
2010	Wheat (cv. Magenta)	Organic matter treatments received oat chaff spread at 20 t ha^{-1}. Both treatment tilled with offset discs.
2011	Wheat (cv. Wyalkatchem)	Both treatment tilled with offset discs.
2012	Canola (cv. Telfer)	Brown manure canola. Organic matter treatments received oat chaff spread at 20 t ha^{-1}. Both treatment tilled with offset discs.
2013	Barley (cv. Hindmarsh)	Both treatment tilled with offset discs.
